# CA 15-3 is predictive of response and disease recurrence following treatment in locally advanced breast cancer

**DOI:** 10.1186/1471-2407-6-220

**Published:** 2006-09-05

**Authors:** Dhafir Al-azawi, Gabrielle Kelly, Eddie Myers, Enda W McDermott, Arnold DK Hill, Michael J Duffy, Niall O Higgins

**Affiliations:** 1 Department of Surgery, St. Vincent's University Hospital, Dublin, Ireland; 2 School of Mathematical Sciences, University College Dublin, Dublin, Ireland; 3 Royal College of Surgeons in Ireland, 123 St. Stephens Green, Dublin, Ireland; 4 Department of Nuclear Medicine, St. Vincent's University Hospital, Dublin, Ireland

## Abstract

**Background:**

Primary chemotherapy (PC) is used for down-staging locally advanced breast cancer (LABC). CA 15-3 measures the protein product of the MUC1 gene and is the most widely used serum marker in breast cancer.

**Methods:**

We retrospectively investigated the role of CA 15-3 in conjunction with other clinico-pathological variables as a predictor of response and time to disease recurrence following treatment in LABC. Pre and post primary chemotherapy serum concentrations of CA 15-3 together with other variables were reviewed and related to four outcomes following primary chemotherapy (clinical response, pathological response, time to recurrence and time to progression). Persistently elevated CA 15-3 after PC was considered as consecutively high levels above the cut off point during and after PC.

**Results:**

73 patients were included in this study. Patients received PC (AC or AC-T regimen) for locally advanced breast cancer. 54 patients underwent surgery. The median follow up was 790 days. Patients with high concentrations of CA 15-3 before PC treatment had a poor clinical (*p *= 0.013) and pathological (*p *= 0.044) response. Together with Her-2/neu expression (*p *= 0.009) and tumour lympho-vascular space invasion (LVI) (*p *= 0.001), a persistently elevated CA 15-3 post PC (*p *= 0.007) was an independent predictive factor of recurrence following treatment in LABC.

**Conclusion:**

Elevated CA 15-3 level is predictive of a poor response to chemotherapy. In addition, persistently elevated CA 15-3 levels post chemotherapy in conjunction with lympho-vascular invasion and HER2 status predict a reduced disease free survival following treatment in locally advanced breast cancer.

## Background

A significant number of women present with locally advanced breast cancer (LABC) despite efforts to promote public awareness. The use of Primary chemotherapy (PC) was initially introduced in patients with LABC, and was first reported in 1978 as part of a multidisciplinary approach [1]. Many studies have subsequently demonstrated the benefits of PC in this setting [2,3]. Much interest has focused on the role of molecular and biological markers in predicting response to PC [4-7], however the precise role of these markers remains controversial. CA 15-3 is a serum-based product of the MUC1 gene and is the most widely used serum marker for breast cancer [8]. Its preoperative levels predict adverse outcome in node negative and node positive breast cancer [9]. Kurebayashi et al have demonstrated that CA 15-3 may have a role in monitoring response to chemotherapy in LABC [10]. This was reinforced by Duffy et al, who suggested that the most important role for CA 15-3 is in monitoring therapy in patients with advanced breast cancer [9,11]. However the precise role of CA15.3 in predicting response to PC and time to disease recurrence in patients with LABC following both PC and surgery is ill defined. Here we provide evidence to support a role for CA15.3 in both conditions.

## Methods

73 patients who presented with LABC between January 1999 and May 2005 were included. All patients received primary chemotherapy in the oncology day clinic at St. Vincent's University Hospital, Dublin, Ireland. All patients were free from metastatic disease at the time of diagnosis as determined by bone scanning, liver ultrasonography and chest x-ray. The histological diagnosis was established either by core biopsy or excisional biopsy. Data was obtained by reviewing the medical records of these patients. The hospital local ethics committee approval was obtained for the study.

### Primary chemotherapy

Earlier patients 48 (65.7%) were treated with 6 cycles of AC (Doxorubicin 60 mg/m^2 ^and Cyclophosphamide 600 mg/m^2 ^at day one and repeated every 21 days). Later patients 25 (34.2%) were treated with 4 cycles of AC followed by 4 cycles of Docetaxol 75 mg/m^2 ^every 21 days).

### Clinical response (CR)

Patients' response to PC was assessed by regular clinical examinations during which the clinical progress was documented at each visit. The UICC criteria were used to assess the clinical response [5]. Patients were divided into complete or partial responders or as having static or progressive disease.

### Surgery

Prior to surgery all patients were regularly assessed for response both clinically and radiologically. All suitable patients underwent surgery within 6–8 weeks after completion of PC. Fifty two patients underwent a modified radical mastectomy and two patients underwent breast conserving surgery. Postoperatively all patients received adjuvant chemotherapy (CMF regimen) and post operative radiotherapy.

### Clinicopathological parameters

Variables analysed include tumour size, histological subtypes, tumour grade, tumour stage, ER and Her-2/neu status. ER status was determined by immunohistochemistry using rabbit antihuman ERα antibody. Her2/neu status was evaluated using the Dako Hercep Test immunocytochemical assay. In tumor samples scoring +2 with the Hercep test, Her-2 status was confirmed by *fluorescent in-situ hybridization *(FISH) test.

### Pathological response (PR)

Chevallier's classification was used to evaluate pathological response to PC [12]. Tumour size, lymph node involvement, evidence of lymphovascular space invasion (LVI), DCIS and tumour response to PC were documented.

### CA15-3 levels

Serum CA 15-3 was measured by ELISA (ES300/Elecsys 2010; Roche Diagnostics). Variation was <6% for commercial control sera (Preci Controls; Roche Diagnostics). CA 15-3 concentrations were stratified using a cut off value of 30 kilo units/L [9,13]. Serum CA 15-3 levels were estimated at the time of diagnosis, following each cycle of PC and every three months following surgery. A persistently elevated CA 15-3 after chemotherapy is defined as consecutive high levels of CA15-3 serum samples (steadily rising or fluctuating levels) above the cut off point at the time of completion of PC during the mentioned follow up period prior to surgery.

### Statistical analysis

SAS version 8.2 statistical program (SAS Institute, Cary, North Carolina, USA) was used in the statistical analysis. The clinical response was re-evaluated as follow: the complete and partial clinical response was considered as a good response while the static and progressive response was considered as a poor response [5]. The pathological response grading was also re-evaluated: Grade I and II were considered as a good PR and grades III and IV as a poor PR [14]. Univariate analysis was performed using Fisher's exact test for categorical variables and Wilcoxon's test for continuous variables. Multivariate analysis was carried out using Cox's proportional hazard model. A *P*-value of less than 0.05 was considered to be significant.

## Results

Seventy three patients received primary chemotherapy for LABC. The mean age was 52.3 years with a range from 26 to 96 years. The mean tumour size was 4.9 cm with a range from 2–10 cm. The median follow up was 790 days. Table 1 illustrates the pathological parameters of tumours in the studied group. The chemotherapeutic regimens used did not influence the outcome.

### Tumour markers

Twenty four patients had high serum levels of CA15-3 prior to chemotherapy. After completion of PC the serum concentrations of CA 15-3 normalised in four patients, one of these patients had bilateral inflammatory breast cancer and did not undergo further surgery. The other three patients underwent surgery and demonstrated a favourable pathological response. All four patients were alive at the end of the study with no disease recurrence. Pre and post chemotherapy CA 15-3 levels did not correlate with any of the clinico-pathological parameters studied (Table 2).

### Clinical response

The seventy three patients were categorised into four groups according the UICC criteria based on response to PC recorded on regular clinical examinations. A complete clinical response was seen in 6 (8.2%) patients, partial clinical response in 38 (52%) patients with no change (stasis) from the original size in 24 (32.8%) patients and progression in 5 (6.8%) patients. Multivariate analysis did not show significance, however univariate analysis showed that tumour stage (*p *= 0.014) and high levels of serum CA15-3 before PC (*p *= 0.013) were significantly associated with a poor clinical response to PC. Table (3) demonstrates the relationship of poor clinical response to the studied clinico-pathological variables.

### Pathological response

Fifty four patients underwent surgery. At the end point of the study, 19 patients were either awaiting surgery or were deemed unsuitable for surgery. Using Chevallier's classification there was a complete pathological response grade I, in 4 (7.5%) patients. Five patients (9.4%) had a grade II response, 21 (39.6%) patients had a grade III response and 24 (45.2%) patients had a grade IV response. Groups I and II were compared with groups III and IV. Multivariate analysis failed to show significant relation between pathological response and other variables. Tumour size, the presence of lympho-vascular space invasion and high levels of serum CA 15-3 before PC (p = 0.001, p = 0.034 and *p *= 0.044 respectively) were significantly associated with an adverse pathological response to PC by univariate analysis. Table (3) demonstrates the relationship of poor pathological response to the clinico-pathological variables studied.

### Time to recurrence

**Figure 1:**
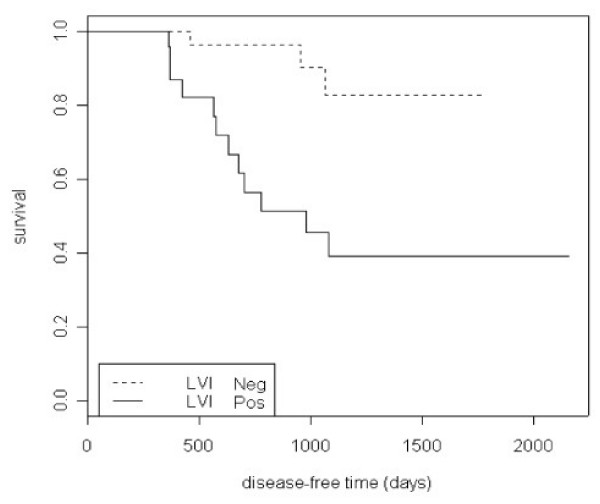
Kaplan-Meiers survival curve shows that presence of LVI is significantly associated with shorter time to recurrence (*p *= 0.001 Cox model).

**Figure 2:**
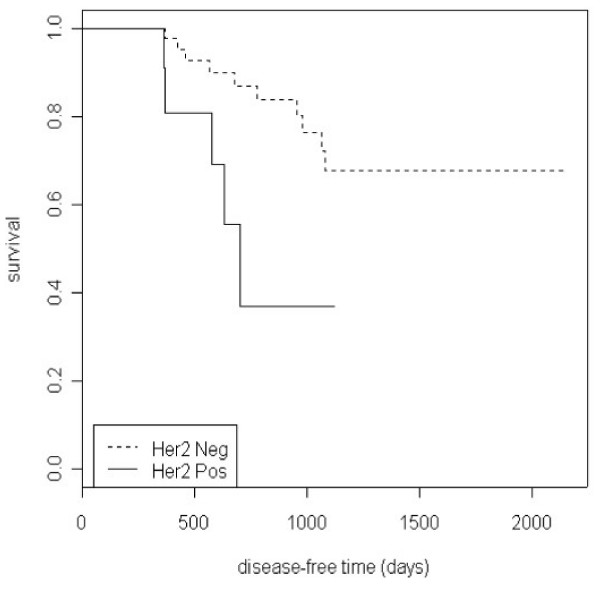
Kaplan-Meiers survival curve, the expression of Her-2/neu receptor was associated with a decrease in disease free time (*p *= 0.009 Cox model).

**Figure 3:**
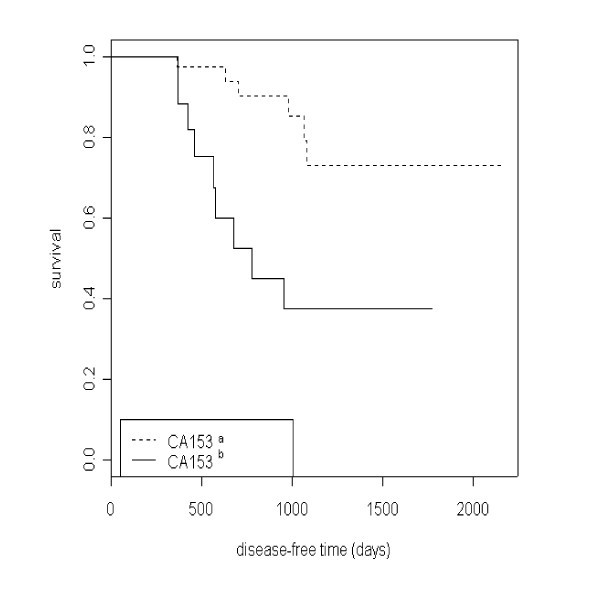
Kaplan-Meiers survival curve shows post PC high serum levels of CA15-3 (CA15-3^a ^levels ≤ 30 KU/L, CA15-3^b ^≥ 30 KU/L) was associated with early disease recurrence (*p *= 0.007 Cox model).

Fifteen patients (20.5%) developed disease recurrence after surgery during the study period; 5 patients developed loco-regional recurrence and 10 patients developed distant organ metastasis. Ten of those who developed recurrence died from breast cancer. The median time to recurrence was 630 days. Six patients of those with disease recurrence had high levels of CA 15-3 before PC while high post PC levels were found in nine patients. Using a multivariate Cox proportional hazard analysis we found that the presence of tumour LVI, Her-2/neu expression and the presence of high serum levels of CA15-3 after PC (*p *= 0.001, *p *= 0.009 and *p *= 0.007 respectively) were three independent risk factors that significantly predict time to disease recurrence after treatment in LABC, figures 1, 2 and 3 respectively.

### Time to progression (TTP)

TTP was defined as the time from the initiation of treatment till the disease progressed or death. Eighteen patients had their disease progressed (15 patients had disease recurrence after surgery and three patients developed disease progression after PC). Using Wilcoxon test high levels of CA 15-3 before PC showed a borderline association with TTP (*P *= 0.0575). However there was a significant relation between high levels of CA 15-3 after PC and TTP (*p *= 0.0001). No association was found between the pathological response and TTP (*P *= 0.3408). However a strong relation was found between the clinical response and TTP (*p *= 0.0001).

## Discussion

CA 15-3 is a serum based product for the MUC1 gene. While CA 15-3 levels have previously been demonstrated to predict outcome in LABC [15]. Its precise role defined by clinical response, pathological response and disease recurrence following PC treatment requires definition. In a recent review Duffy et al concluded that the most important application of CA 15-3 is in monitoring therapy in patients with advanced breast cancer [11]. Expert panels such as NACB and EGTM recommend the routine use of CA 15-3 in monitoring therapy in patients with LABC [16,17]. In contrast the routine use of CA 15-3 in LABC is not recommended by the ASCO panel [18]. Here we provide evidence to support a role for CA 15-3 in LABC.

Much interest has focused on predictors of response to treatment in LABC. Chang et al and Mauriac et al [19,20] showed a favourable outcome in ER negative patients following treatment for LABC. We found no relation between ER and clinical response however a borderline relation was found between ER positive patients and poor pathological response *p *= 0.052 (table 3), a larger sample of patients may be needed to show a significant relation. However these markers all require tumour tissue. CA 15-3 has the advantage of being a serum based marker and therefore its measurement is less invasive. Kurebayashi et al demonstrated that in pre-treatment CA15-3 positive patients changes in CA 15-3 levels after systemic therapy correlated with response to treatment [10]. Here we show that a high CA 15-3 level before PC along with advanced tumour stage predict a poor clinical response to chemotherapy. A poor pathological response to chemotherapy is predicted by elevated pre chemotherapy CA 15-3 levels, large tumour size and the presence of lympho-vascular invasion. Kurebayashi et al also demonstrated that a greater than 20% reduction in CA 15-3 levels following treatment was a favourable predictive factor for time to disease progression during systemic therapy. In this study we found that post chemotherapy high CA 15-3 levels were associated with reduced time to disease progression. We used a Cox proportional hazards model to identify predictors of recurrence following both chemotherapy and surgery in LABC. Detection of conventional markers such as tumour size, lympho-vascular invasion and hormone receptor status in addition to more recent parameters such as HER2, KI-67 and p53 have all been associated with outcome in LABC [19,20,4]. We found that lympho-vascular invasion, HER2 status and elevated CA15-3 levels post chemotherapy were independent predictors of a poor outcome. De La Lande et al reported a relation between CA 15-3 lead time and prognosis [21]. In this study we report a predictive value of high CA 15-3 levels in LABC following PC in relation to TTP and recurrence after surgery. These findings may have important implications as they suggest that surgery may be of minimal benefit in patients who have persistently elevated CA15-3 levels following chemotherapy in LABC. It is not surprising that HER2 status is an independent predictor of poor outcome and this subgroup of patients would undoubtedly benefit from Herceptin.

## Conclusion

Elevated CA 15-3 levels predict a poor response to primary chemotherapy in locally advanced breast cancer. In addition persistent elevation of CA 15-3, post chemotherapy, in conjunction with the presence of lympho-vascular invasion and HER2 positivity predict a reduced disease free survival following treatment in locally advanced breast cancer.

## Competing interests

The author(s) declare that they have no competing interests.

## Authors' contributions

DA carried out data collection, designed the study and drafted the manuscript. GK participated in the design of the study and performed the statistical analysis. EM participated in the design of the study and helped to draft the manuscript. EW participated in its design, coordination and helped to draft the manuscript. AH conceived of the study, participated in its design, coordination and helped to draft the manuscript. All authors read and approved the final manuscript.

## Pre-publication history

The pre-publication history for this paper can be accessed here:



## Figures and Tables

**Table 1:** Pathological parameters of the tumours in the investigated group.

	Number	%
Inflammatory carcinoma	24	32.8
Tumour type		
Ductal	61	83.5
Lobular	8	10.9
Mixed	4	5.4
Tumour grade		
I	1	1.37
II	27	36.9
III	45	61.6
LVI	26	35.6
Stage of the tumour		
2	11	15
3	60	82.1
4	2 (SCL)	2.7

SCL (supraclavicular lymph nodes)

**Table 2:** The relation of high CA 15-3 concentration with the clinico-pathological variables analyzed.

	**High CA 15-3 before PC**	***p *value**	**High CA 15-3 after PC**	***p *value**
**Tumour size**				
**<2.5 cm**	2/24(8.3%)		2/22(9.1%)	
**>2.5 cm**	22/24(91.6%)	0.627	20/22(90.9%)	0.884
**Tumour histology**				
**Ductal**	19/24(79.1%)	0.533	18/22(81.8%)	0.875
**Lobular**	4/24(16.6%)		3/22(13.6%)	
**Mixed**	1/24(4.1%)		1/22(4.5%)	
**Tumour stage**				
**II**	2/24(8.3%)		1/22(4.5%)	
**III**	20/24(83.3%)	0.266	20/22(90.9%)	0.256
**IV**	2/24(8.3%)		1/22(4.5%)	
**Her2/neu positive**	5/24 (20.8%)	0.487	5/22(22.7%)	1.000
**ER positive**	14/24(58.3%)	1.000	12/22(54.5%)	0.796
**LVI**	10/24(41.6%)	0.603	11/22(50%)	0.114

Fisher's exact test was used for categorical variables and Wilcoxon's test for continuous variables. A *P*-value of less than 0.05 was considered to be significant. LVI (lympho-vascular space invasion) and PC (primary chemotherapy).

**Table 3:** The relation of poor clinical and pathological response to the clinico-pathological variables.

	**Poor clinical Response**	***p *value**	**Poor pathological Response**	***p *value**
**Tumour size**				
**<2.5 cm**	1/29(3.4%)		5/45(11%)	
**>2.5 cm**	28/29(96.5%)	0.052	40/45(88.8%)	0.001
**Tumour Histology**				
**Ductal**	26/29(89.6%)	0.216	40/45(74%)	0.187
**Lobular**	1/29(3.4%)		2/45(3.7%)	
**Mixed**	2/29(6.8%)		3/45(5.5%)	
**Tumour stage**				
**II**	2/29(6.8%)		8/45(17.7%)	0.26
**III**	24/29(82.7%)	0.014	37/45(82.2%)	0.26
**IV**	3/29(10.3%)		Non	
**Her2/neu positive**	8/29(27.5%)	0.410	8/45(17.7%)	0.666
**ER positive**	18/29(62.1%)	0.396	32/45(91.4)	0.052
**LVI**	13/29(44.8%)	0.141	23/45(51.1%)	0.034
**CA 15-3 before PC**	13/29(50%)	0.013	17/45(37.7%)	0.044
**CA 15-3 after PC**	14/29(46.2%)	0.012	16/45(35.5%)	0.045

Fisher's exact test was used for categorical variables and Wilcoxon's test for continuous variables. A *P*-value of less than 0.05 was considered to be significant. LVI (lympho-vascular space invasion) and PC (primary chemotherapy).
